# Incidence of low-triiodothyronine syndrome in patients with septic shock

**DOI:** 10.5935/0103-507X.20200088

**Published:** 2020

**Authors:** Matias German Cornu, Andrés Luciano Nicolas Martinuzzi, Pedro Roel, Laura Sanhueza, Mariana Elisabeth Sepúlveda, Martin Sergio Orozco, Carlos Arturo Sánchez, Melina Gulino

**Affiliations:** 1 Intensive Care Unit, Centro de Medicina Integral del Comahue - Neuquén, Argentina.

**Keywords:** Sepsis, Infection, Hemodynamics, Metabolism, Prognosis, Sepsis, Infecciones, Hemodinámica, Metabolismo, Prognóstico

## Abstract

**Objective:**

Low levels of thyroid hormones have been associated with poor clinical outcomes. This metabolic situation, designated euthyroid sick syndrome, has been interpreted as a state of adaptation to different pathological processes, characterized by the decrease in plasma triiodothyronine. The present study seeks to determine the incidence of this disorder in patients with septic shock and its relationship with other severity indices and clinical outcomes.

**Methods:**

This prospective analytical study evaluated patients admitted to the intensive care unit with septic shock between April 2018 and July 2019. Variables associated with septic shock and thyroid profile were recorded at the time of the septic shock diagnosis and 7, 14, and 21 days later.

**Results:**

A total of 27 patients who met the inclusion criteria were analyzed. The incidence of an altered thyroid axis was 96.3%, with a mortality at 28 days of 36.7%. Patients without hormonal alteration did not present negative outcomes. Among those with low triiodothyronine, 42.3% recovered their thyroid function within 28 days, in whom mortality was 0%; 57.7% did not recover their thyroid function, in whom mortality was 66.7%. Those whose thyroid axis was altered and who did not normalize its function required more doses of vasoactives and had deteriorated lactate clearance.

**Conclusion:**

Patients with septic shock have a high incidence of alteration of the thyroid axis, and this dysfunction is associated with higher mortality.

## Abstract (es)

**Objetivo:**

Se ha visto asociación entre los bajos niveles de hormonas tiroideas y malos resultados clínicos. Esta situación metabólica designada bajo el término de enfermedad eutiroidea, ha sido interpretada como un estado de adaptación a diferentes procesos patológicos, caracterizada por la disminución plasmática de triiodotironina T3. El presente estudio busca determinar la incidencia de este trastorno en los pacientes con shock séptico y su relación con otros índices de gravedad, y resultados clínicos.

**Métodos:**

Estudio de corte prospectivo analítico, evaluó a los pacientes que ingresaron con shock séptico a la unidad de terapia intensiva, durante el periodo abril 2018 - julio 2019. Se registraron variables asociadas al shock séptico, y el perfil tiroideo al momento del diagnóstico de shock séptico, a los 7, 14 y 21 días.

**Resultados:**

Se analizaron 27 pacientes que cumplieron con los criterios de inclusión. La incidencia de alteración del eje tiroideo fue del 96,3%s, con una mortalidad a los 28 días de 36,7%. Los pacientes sin alteración hormonal no presentaron desenlaces negativos. Entre los que presentaron baja triiodotironina, 42,3% recupero la función tiroidea dentro de los 28 días, con mortalidad del 0%. No recuperaron función tiroidea (57,7%), con una mortalidad del 66,7%. Comparativamente se observó que aquellos que presentaron alteración del eje y no normalizaron la función, requirieron más dosis de vasoactivos, y deterioro del *clearence* de lactato.

**Conclusión:**

Los pacientes con shock séptico presentan una alta incidencia de alteración del eje tiroideo y esta disfunción se asoció a mayor mortalidad.

## INTRODUCTION

In critically ill patients, it is common to observe low concentrations of thyroid hormones. This hormonal imbalance is known as euthyroid sick syndrome.^(1,2)^ It is characterized by low serum triiodothyronine (T_3_) and normal or low levels of thyroid-stimulating hormone (TSH), increases in serum reverse T_3_, and decreased thyroxine (T_4_). This syndrome is described as an adaptive mechanism to stress that seeks to reduce energy requirements. Some publications question this hypothesis, stating that this alteration has a role in the pathophysiology of critical illness and especially in septic states.^(3)^

Septic shock is a disease state characterized by an inflammatory response to infection that triggers a state of tissue hypoperfusion, and it is associated with high mortality. Its pathophysiology involves a profound hemodynamic deterioration, characterized by vasoplegia that can be associated with deteriorated myocardial function, together with different degrees of alterations at the microcirculatory level, with cytopathic lesions up to failure of mitochondrial respiration (mitochondrial and microcirculatory distress syndrome). In this context, it could be interpreted that the actions of T_3_ are part of the pathophysiological mechanism of septic shock.

The cardiovascular system and oxygen metabolism at the mitochondrial level are some of the most important targets of thyroid hormones. Triiodothyronine is the metabolically active hormone that is produced by peripheral conversion of 80% of the T_4_ secreted by the thyroid gland. Triiodothyronine plays a fundamental role in modulating cardiovascular performance and mitochondrial activity.^(4-6)^ The actions of this hormone are produced by genomic mechanisms (actin and myosin synthesis, adrenergic receptors, etc.) as well as nongenomic mechanisms (Ca^+^ modulation) on the cardiovascular system, improving cardiac output and increasing blood flow to tissues. Additionally, by means of mitochondrial T_3_ receptors, a direct effect on these organelles is produced, increasing the production and use of adenosine triphosphate and increasing metabolism and oxygen consumption.

The main pathophysiological mechanism involved in low-T_3_ syndrome (LT_3_S) is low activity of the 5′ monodeionidase enzyme that converts T_4_ to T_3_ in peripheral tissues.^(7-9)^ In septic states, pro-inflammatory cytokines (interleukin - IL - 1, IL-6, tumor necrosis factor alpha - TNF-α) perturb thyroid hormone levels by decreasing the activity of type I deiodinase, thereby decreasing the peripheral conversion of T_4_ to T_3_.^(10-15)^

The primary objective of this study was to measure the incidence of LT_3_S in our population of patients with septic shock. The secondary objectives were to analyze its impact on prognosis and its relationship with other severity indices associated with septic shock.

## METHODS

This analytical prospective cohort study was designed to evaluate the incidence of impaired thyroid function in patients with septic shock during admission to the intensive care unit (ICU). The period of enrollment and follow-up of patients was April 1, 2018 to June 30, 2019. The patients admitted to the study were followed up until their institutional discharge.

Patients older than 18 years who were admitted consecutively to the ICU and who met the inclusion criteria were included. Septic shock was diagnosed according to the Third Consensus Conference SEPSIS-3.^(16)^ In this conference, sepsis was defined as infection associated with organ failure, which in turn was defined as a rise in the Sequential Organ Failure Assessment (SOFA) score greater by ≥ 2 points from baseline. In addition, septic shock was defined as sepsis with persistent hypotension that requires vasoactive to achieve mean arterial pressure ≥ 65mmHg and serum lactate ≥ 2mmol/L after adequate volume resuscitation. Patients with a history of thyroid disease (hypo/hyper/tumor) or with a previous diagnosis of endocrine abnormality (thyroid or adrenal axis) were excluded, and patients under treatment with amiodarone or any other alteration that could perturb the thyroid hormone profile of the patient were excluded.

The general demographic data of sex, age, date of admission to the ICU, reason for admission, diagnosis of sepsis, and suspected/probable focus were collected. The Charlson comorbidity score, Acute Physiology and Chronic Health Evaluation II (APACHE II) score, and SOFA score. Thyroid hormone profile with dosages every 7 days until day 21 of diagnosis (T0, T7, T14, T21) was as described in table 1. Likewise, serum lactate, hemodynamic parameters, and the requirement of vasoactive drugs and their doses were recorded. Length of ICU stay and mortality at 28 days were recorded.

**Table 1:** Laboratory method and thyroid profile values

Thyroid profile	Measurement	Laboratory ranges	Method
TSH	At SS diagnosis, days 7, 14, and 21	0.27 - 4.200*µ*U/mL	Roche© Electrochemiluminescence
T_4_ free	At SS diagnosis, days 7, 14, and 21	0.93 - 1.70ng/dL	COBAS e411 analyzer
T_4_ total	At SS diagnosis, days 7, 14, and 21	5.10 - 14.10*µ*g/dL	
T_3_	At SS diagnosis, days 7, 14, and 21	80 - 200ng/dL	

TSH - thyroid-stimulating hormone; SS - septic shock; T_4_ - thyroxine; T_3_ - triiodothyronine.

Patients were first divided into two groups according to the presence or absence of LT_3_S, considered in this study as T_3_ below 80ng/dL. Then, patients who presented LT_3_S were subdivided into those whose low T_3_ persisted throughout the study period and those who normalized their T_3_ in the same period.

This information was recorded in the AVICENNA program and was then reduced to an Excel Microsoft® workbook to be incorporated into the statistical analysis program.

### Statistical analysis

The data collected were analyzed statistically using IBM Statistical Package for Social Science (SPSS®) 25 software. The data were condensed to location (mean), dispersion (standard deviation), and aggregation (percentages), respectively. The existence of differences between the means, proportions, and results was evaluated using comparison tests of proportions based on the Z distribution. A cutoff of 5% was chosen for significance. Survival was analyzed using the Kaplan-Meier nonparametric test.

## RESULTS

**Figure 1:**
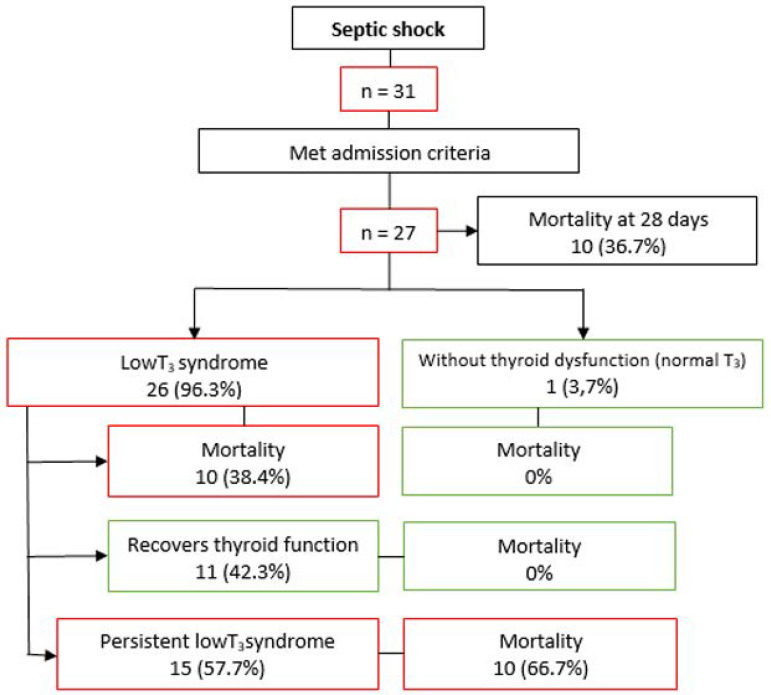
Patient flowchart. The mortality expressed in the figure is at 28 days. T_3_ - triiodothyronine.

During the study period, 31 patients meeting the diagnostic criteria for septic shock were recruited; in 27 of these, all the procedures designed for the study were completed (Figure 1). Table 2 shows the baseline data of these patients. The mean concentrations of thyroid hormones are given in table 3. Of the patients included in the study, 96.3% (n = 26) had LT_3_S. Of these, 42.3% (n = 11) recovered thyroid axis function within 28 days of follow-up (Table 4).

**Table 2:** Clinicodemographic data of the whole population, survivors, and nonsurvivors

	Sample total n = 27	Survivors n = 17	Nonsurvivors n = 10	p value
Age (years)	55.9 ± 16	56 ± 19	55..9 ± 11	0.98
Sex, female	51.9	60	36.4	0.24
APACHE II	16.2 ± 6.6	14.9 ± 6.22	18..5 ± 6.9	0.17
SOFA	9.7 ± 3.6	8.9 ± 2.96	11.2 ± 4.3	0.11
Kidney failure	15 (55.6)	7 (41.2)	8 (80.0)	0.054
AKI I	4 (26.6)	3 (42.8)	1 (12.5)	0.2
AKI II	3 (20)	2 (28.6)	1 (12.5)	0.45
AKI III	8 (53.4)	2 (28.6)	6 (75.0)	0.082
Focus				
Abdominal	55.6	64.7	40.0	0.32
Respiratory	18.5	11.8	30.0	0.248
Urinary	14.8	11.8	20.0	0.57
Endovascular	3.7	5.9		NC
Other	7.4	5.9	10.0	0.7
Charlson index	3.6 ± 2.7	3 ± 2.85	4.7 ± 2.31	0.12
Days of hospitalization	19.5 ± 7.6	24 ± 22	11.9 ± 8.19	0.11
Mortality 28 days	10 (36.7)	NC	NC	NC
Thyroid dysfunction	26 (96.3)	16 (94.1)	10 (100)	0.44
Recovered function	44.4	70.6	0	0.0005

APACHE II - Acute Physiology and Chronic Health Evaluation II; SOFA - Sequential Organ Failure Assessment; AKI - acute kidney injury; NA - not applicable; 95%CI - 95% confidence interval. Results expressed as mean ± standard deviation, %, or n (%).

**Table 3:** Thyroid profile of survivors versus nonsurvivors

	Survivors (n = 17)	Nonsurvivors (n = 10)	p value
TSH at diagnosis	1.02 ± 0.98	3.05 ± 4.83	0.102
TSH day 7	4.98 ± 3.06	3.77 ± 5.73	0.47
TSH day 14	3.84 ± 2.05	8.64 ± 14.80	0.18
TSH day 21	3.19 ± 2.11	3.63	NC
T_3_ at diagnosis	52.06 ± 17.83	48.62 ± 17.40	0.53
T_3_ day 7	69.81 ± 21.09	52.09 ± 17.19	0.033
T_3_ day 14	96.07 ± 30.12	41.89 ± 10.68	0.0009
T_3_ day 21	72.33 ± 16.99	55.60	NC
T_4_ at diagnosis	5.01 ± 1.76	4.15 ± 1.33	0.19
T_4_ day 7	5.72 ± 1.31	3.54 ± 2.18	0.0032
T_4_ day 14	7.24 ± 1.33	3.41 ± 1.97	0.0001
T_4_ day 21	6.20 ± 1.30	3.34	NC

TSH - thyroid-stimulating hormone; T_3_ - triiodothyronine; T_4_ - thyroxine; NA - not applicable. Results expressed as mean ± standard deviation or absolute value.

**Table 4:** Comparison of recovered thyroid function versus persistent low T_3_ syndrome

	Recovery of T_3_ plasma level n = 11	Persistent low T_3_ syndrome n = 15	p value
Age (years)	56.8 ± 20.6	55.4 ± 16	0.84
Sex, female	50.0	53.3	0.87
APACHE II	13.6 ± 5.22	18.3 ± 7	0.07
SOFA	8.8 ± 3	10.5 ± 3.9	0.23
Focus			
Abdominal	75	40	0.07
Respiratory	8.3	26.7	0.23
Urinary	8.3	20	0.41
Endovascular		6.7	NC
Other	8.3	6.7	0.87
Charlson index	2.8 ± 2.6	4.2 ± 2.7	0.19
Days of hospitalization	15.6 ± 5.7	13.8 ± 13.5	0.68
Mortality 28 days	0	66.7%	0.013
T_3_ at the diagnosis of shock (ng/dL)	53.15 ± 13.7	48.9 ± 20	0.54
T_3_ day 7	73.9 ± 20	54.4 ± 17.9	0.015
T_3_ day 14	99.8 ± 28	44.6 ± 11.7	0.0001
T_3_ day 21	73.2 ± 20	62.5 ± 9.9	0.084
Lactate 0 hour (mmol/L)	5.4 ± 4.1	3.04 ± 3.4	0.12
Lactate 24 hours (mmol/L)	3.1 ± 2.7	3.5 ± 4.4	0.79
Clearance lactate 24 hours	42	-15	0.0046
Lactate 48 hours (mmol/L)	2.4 ± 1.6	2.3 ± 1.1	0.85
Lactate 72 hours (*µ*/kg/min)	1.7 ± 0.9	2.22 ± 1.58	0.33
Clearance lactate 72 hours	68.7%	36.5%	0.11
NDL day 1 (*µ*/kg/min)	0.31 ± 0.26	0.79 ± 0.58	0.017
NDL day 2 (*µ*/kg/min)	0.46 ± 0.38	0.63 ± 0.6	0.41
NDL day 3 (*µ*/kg/min)	0.14 ± 0.05	0.55 ± 0.68	0.058
NDL day 4 (*µ*/kg/min)	0.05 ± 0.05	0.68 ± 0.7	0.0068

T_3_ - triiodothyronine; APACHE II - Acute Physiology and Chronic Health Evaluation II; SOFA - Sequential Organ Failure Assessment; NA - not applicable; OR - odds ratio; 95%CI - 95% confidence interval NDL - noradrenaline. Results expressed as mean ± standard deviation or %.

**Figure 2:**
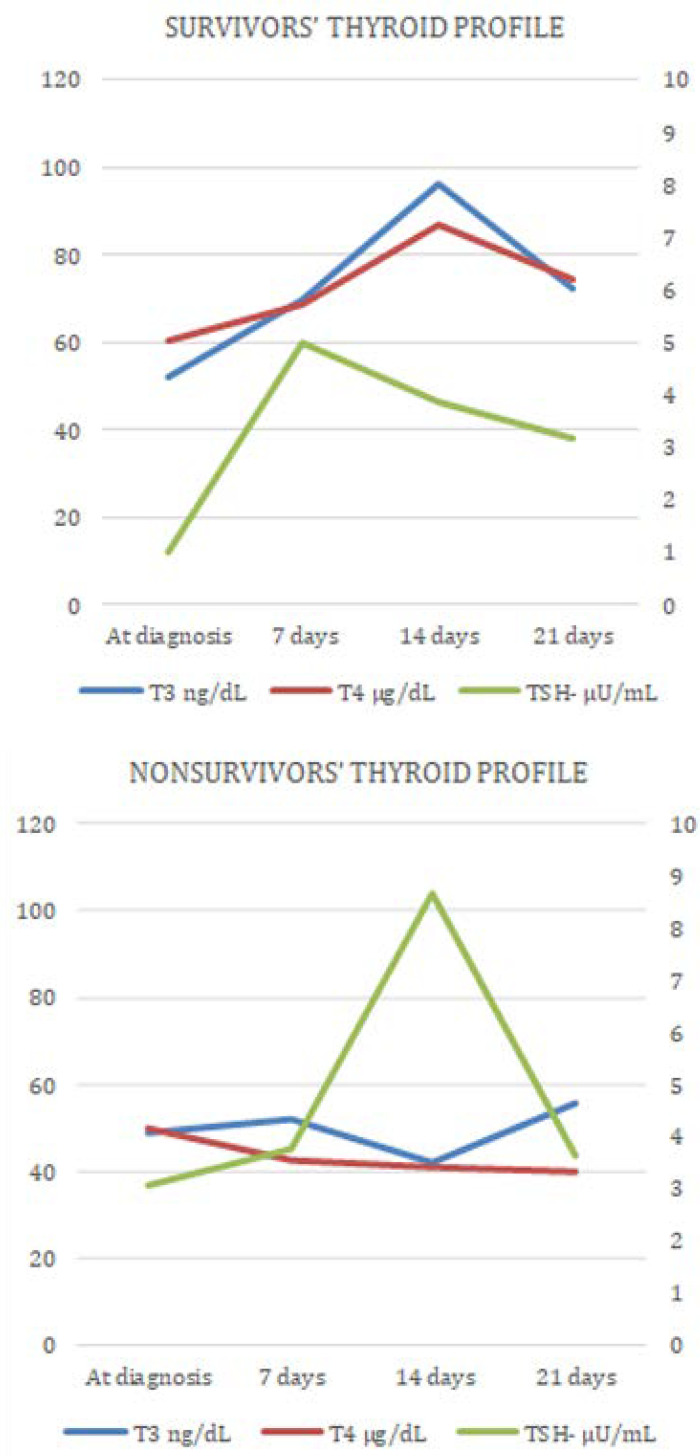
Thyroid profile behavior in survivors versus nonsurvivors. T_3_ - triiodothyronine; TSH - thyroid-stimulating hormone; T4 - thyroxine.

Triiodothyronine was lower in those who died than those who survived (admission 53.15ng/dL ± 13.7 *versus* 48.9ng/dL ± 20, p = 0.54; 7th day 73.9ng/dL ± 20 *versus* 54.4ng/dL ± 17.9, p = 0.015; 14th day 99.8ng/dL ± 28 *versus* 44.6ng/dL ± 11.7, p = 0.0001; 21st day 73.2ng/dL ± 20 *versus* 62.5 ng/dL ± 9.9, p = 0.084), associated with the changes to the rest of the thyroid axis (T_4_, TSH). See figure 2, where the dynamic behavior of thyroid hormone values (TSH, T_3_, T_4_) during the follow-up is compared between survivors and nonsurvivors.

Patients who normalized their thyroid function within the study period and those who persisted with LT_3_S did not show statistically significant differences in age, sex, or APACHE II, SOFA, or Charlson comorbidity index score (Table 2). A difference was observed in the infectious focus: those who normalized their thyroid function had a higher incidence of abdominal sepsis, but without being statistically significant or suggesting any inferences we could make.

The T_4_ values in the patients who survived remained within normal values throughout the study time, while nonsurvivors had low, progressively decreasing T_4_ (admission 5.01µg/dL ± 1.76 *versus* 4.15µg/dL ± 1.33, p = 0.19; 7th day 5.72µg/dL ± 1.3 *versus* 3.54µg/dL ± 2.18, p = 0.0032; 14th day 7.24µg/dL ± 1.33 *versus* 3.41µg/dL ± 1.97, n = 5; 21st day 6.20µg/dL ± 1.30 *versus* 3.34, n = 1). Similarly, TSH was normal in the patients who survived, but in patients who died, a peak TSH stimulation was observed at day 14. The thyroid profile in its entirety could not be adequately analyzed at day 21 because the patients who died did so before this date, so there were hormonal values in only one case.

When hemodynamic parameters were compared between both groups, only vasoactive drug requirements at admission were higher in patients who died than those who survived (0.79 ± 0.58 *versus* 0.31 ± 0.26; p = 0.017); see figure 3A. In both groups, after initial resuscitation, lactate levels were modified. In the persistent LT_3_S group, lactate stayed above 2mmol/L, whereas the group that normalized thyroid function had lactate that decreased to normal at 72 hours after admission (Figure 3B).

**Figure 3A:**
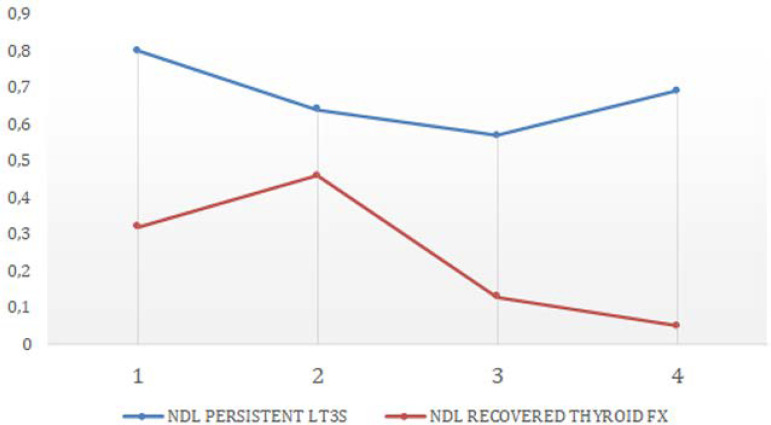
Differences in noradrenaline requirements between patients with persistent low T_3_ syndrome and those who recovered their T_3_ level. NDL - noradrenaline; LT3S - low T_3_ syndrome; FX - function.

**Figure 3B:**
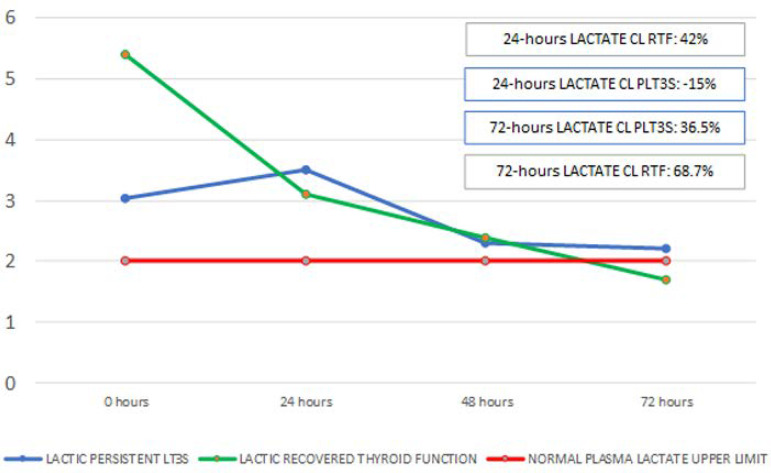
Differences in the behavior of plasma lactate between patients with persistent low T_3_ syndrome and those who recovered their T_3_ level. PLT_3_S - persistent low T_3_ syndrome; FX - function; CL - clearance; RTF - recovers thyroid function.

When the incidence of kidney failure related to thyroid disorder was analyzed, there were no statistically significant differences between the two groups, although there was a tendency to see more kidney failure in the group of nonsurvivors (Table 2).

**Figure 4:**
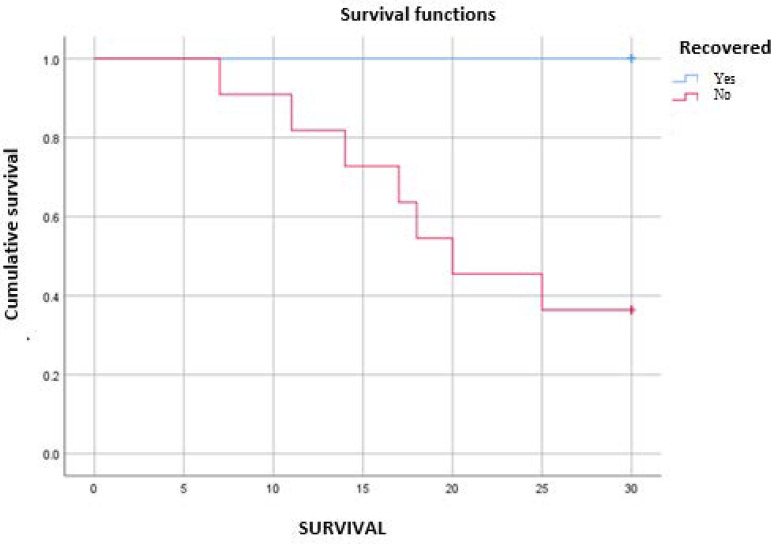
Persistent low-T3 syndrome survival curve versus recovery of thyroid function.

The total mortality of the sample at 28 days was 36.7%. When mortality was analyzed according to the presence of thyroid dysfunction, those who normalized their thyroid function had a mortality of 0%, compared with 66.7% among the patients who did not normalize their thyroid function within 28 days (Figure 1). Figure 4 shows the Kaplan-Meier survival curve, which shows a difference in mortality at 28 days.

## DISCUSSION

The present study evaluated the relationship between low T_3_ and septic shock and the probable relationship of low T_3_ with disease severity.

Thyroid hormones play an important role in the hemodynamic and metabolic functions of the body, among others. In the course of different disease states, the levels of T_3_, T_4_, and TSH decrease, a phenomenon called euthyroid sick syndrome or nonthyroidal illness syndrome.^(17)^ These changes in circulating hormone levels are proportionally associated with disease severity and survival.

Neuroendocrine dysfunction is common in critically ill patients, including septic states,^(18-20)^ and it has been described as an independent predictor of mortality in this group of critically ill patients, as well as in other pathologies of similar severity.^(21-24)^ Likewise, studies carried out on brain-dead patients during body maintenance show that more organs can be harvested for transplantation from those who undergo hormonal resuscitation therapy, with supplementation of thyroid hormones, corticosteroids, and insulin, with the objective of generating greater hemodynamic stability.^(25-28)^

The levels of thyroid hormones are lower in septic patients than nonseptic patients with equal severity of disease, and low T_3_ can contribute to associated organ dysfunction; these alterations are associated with worse evolution.^(19)^

The present study shows the high incidence of impaired thyroid function in this group of patients and shows a relationship between the severity of septic patients and thyroid axis dysfunction. Patients with persistent deterioration of hormone levels (T_3_, T_4_) during the study period had higher mortality than those who normalized the function of the thyroid axis.

There was also evidence of greater hemodynamic deterioration as evaluated by SOFA (vasoactive drug requirement) (Figure 3A) and lower lactate clearance associated with thyroid deterioration (Figure 3B). This might have been associated with the deterioration of the physiological effects of thyroid hormones on cardiovascular performance (hemodynamic effects), as well as their metabolic actions on oxygen metabolism at the mitochondrial level.

On this basis, it could be hypothesized that LT_3_S in patients with septic shock is part of the pathophysiology of this disease and/or an associated organ (endocrine-metabolic) failure and not just an adaptive phenomenon. Therefore, substitution treatment with synthetic thyroid hormones could modify the hemodynamic symptoms of septic patients, contributing in part to the decrease in their morbidity and mortality. There are few publications, either experimental and clinical, on this topic, particularly in this group of patients, and they have had small samples and some contradictory results.^(17)^

As noted above, the results of this study, despite coming from a small sample, reflect a probable association between LT_3_S and septic shock severity and mortality at 28 days. The role of hormone replacement therapy in septic patients with LT_3_S, and therefore its positive or negative effect on the clinical course of the disease, remains to be clarified.

## CONCLUSION

Patients with septic shock have a high incidence of altered thyroid axis, characterized by low T_3_. In this study, hormonal alterations were more severe and persistent in patients who died, suggesting an association between low T_3_ and mortality. T_4_ behaved similarly. Persistently low T_3_ was associated with higher vasoactive requirements and deterioration of lactate clearance.

In summary, low-T_3_ syndrome had a high incidence in our series of patients with septic shock, and this dysfunction was a marker of severity in this specific group of patients. The limitations of our study are that it was done in a single center in a small sample, precluding a multivariate analysis to assess thyroid alteration as an independent predictor of mortality.
